# Diagnostic performance of IVUS-FFR analysis based on generative adversarial network and bifurcation fractal law for assessing myocardial ischemia

**DOI:** 10.3389/fcvm.2023.1155969

**Published:** 2023-03-20

**Authors:** Dong Yong, Chen Minjie, Zhao Yujie, Wang Jianli, Liu Ze, Li Pengfei, Lai Xiangling, Liu Xiujian, Del Ser Javier

**Affiliations:** ^1^ Department of Cardiology, the 7th People’s Hospital of Zhengzhou, Zhengzhou, China; ^2^ School of Biomedical Engineering, Sun Yat-sen University, Shenzhen, China; ^3^ TECNALIA, Basque Research & Technology Alliance (BRTA), Derio, Spain; ^4^ University of the Basque Country (UPV/EHU), Bilbao, Spain

**Keywords:** computational fluid dynamics, coronary blood flow, bifurcation fractal law, generative adversarial network, coronary artery disease, side-branch blood flow, intravascular ultrasound (IVUS)

## Abstract

**Background:**

IVUS-based virtual FFR (IVUS-FFR) can provide additional functional assessment information to IVUS imaging for the diagnosis of coronary stenosis. IVUS image segmentation and side branch blood flow can affect the accuracy of virtual FFR. The purpose of this study was to evaluate the diagnostic performance of an IVUS-FFR analysis based on generative adversarial networks and bifurcation fractal law, using invasive FFR as a reference.

**Method:**

In this study, a total of 108 vessels were retrospectively collected from 87 patients who underwent IVUS and invasive FFR. IVUS-FFR was performed by analysts who were blinded to invasive FFR. We evaluated the diagnostic performance and computation time of IVUS-FFR, and compared it with that of the FFR-branch (considering side branch blood flow by manually extending the side branch from the bifurcation ostia). We also compared the effects of three bifurcation fractal laws on the accuracy of IVUS-FFR.

**Result:**

The diagnostic accuracy, sensitivity, and specificity for IVUS-FFR to identify invasive FFR≤0.80 were 90.7% (95% CI, 83.6–95.5), 89.7% (95% CI, 78.8–96.1), 92.0% (95% CI, 80.8–97.8), respectively. A good correlation and agreement between IVUS-FFR and invasive FFR were observed. And the average computation time of IVUS-FFR was shorter than that of FFR-branch. In addition to this, we also observe that the HK model is the most accurate among the three bifurcation fractal laws.

**Conclusion:**

Our proposed IVUS-FFR analysis correlates and agrees well with invasive FFR and shows good diagnostic performance. Compared with FFR-branch, IVUS-FFR has the same level of diagnostic performance with significantly lower computation time.

## 1. Introduction

Accurately diagnosing coronary artery stenosis is important in guiding the clinical management of patients with known or suspected ischemic heart disease (1, 2). The evaluation methods for coronary stenosis include morphological evaluation and functional evaluation. Identifying information on these two aspects can accurately guide percutaneous coronary intervention (PCI). For morphological evaluation and functional evaluation, respectively, IVUS and invasive FFR are currently regarded as the gold standards in clinical practice (3–5). However, neither IVUS nor invasive FFR alone can comprehensively evaluate coronary artery stenosis. IVUS alone is difficult to determine the impact of vascular stenosis on distal coronary blood flow, and cannot accurately evaluate the relationship between stenosis and myocardial ischemia (6). invasive FFR alone cannot determine the type of vascular plaque, stenosis location and size, and other morphological information. Therefore, it is necessary to combine the morphological information provided by IVUS and the functional information provided by invasive FFR to precisely guide PCI.

The clinical feasibility of performing both IVUS and invasive FFR on the same patient is low. Both IVUS and invasive FFR are interventional exams with the traits of being high risk and requiring a lot from the operator (7, 8). Performing IVUS and invasive FFR at the same time will prolong the operation time and increase the risk of surgery. In addition, performing two tests on the same patient can significantly increase the cost of inspections (9). In response to the above problems, researchers proposed a virtual FFR analysis method relying on IVUS images. This method provides a way to comprehensively evaluate coronary stenosis from both morphological and functional aspects with only one IVUS examination. Multiple studies have demonstrated favorable diagnostic accuracy for this method compared to invasive FFR and can reduce surgical risks and inspection costs (10–14).

**Figure 1:**
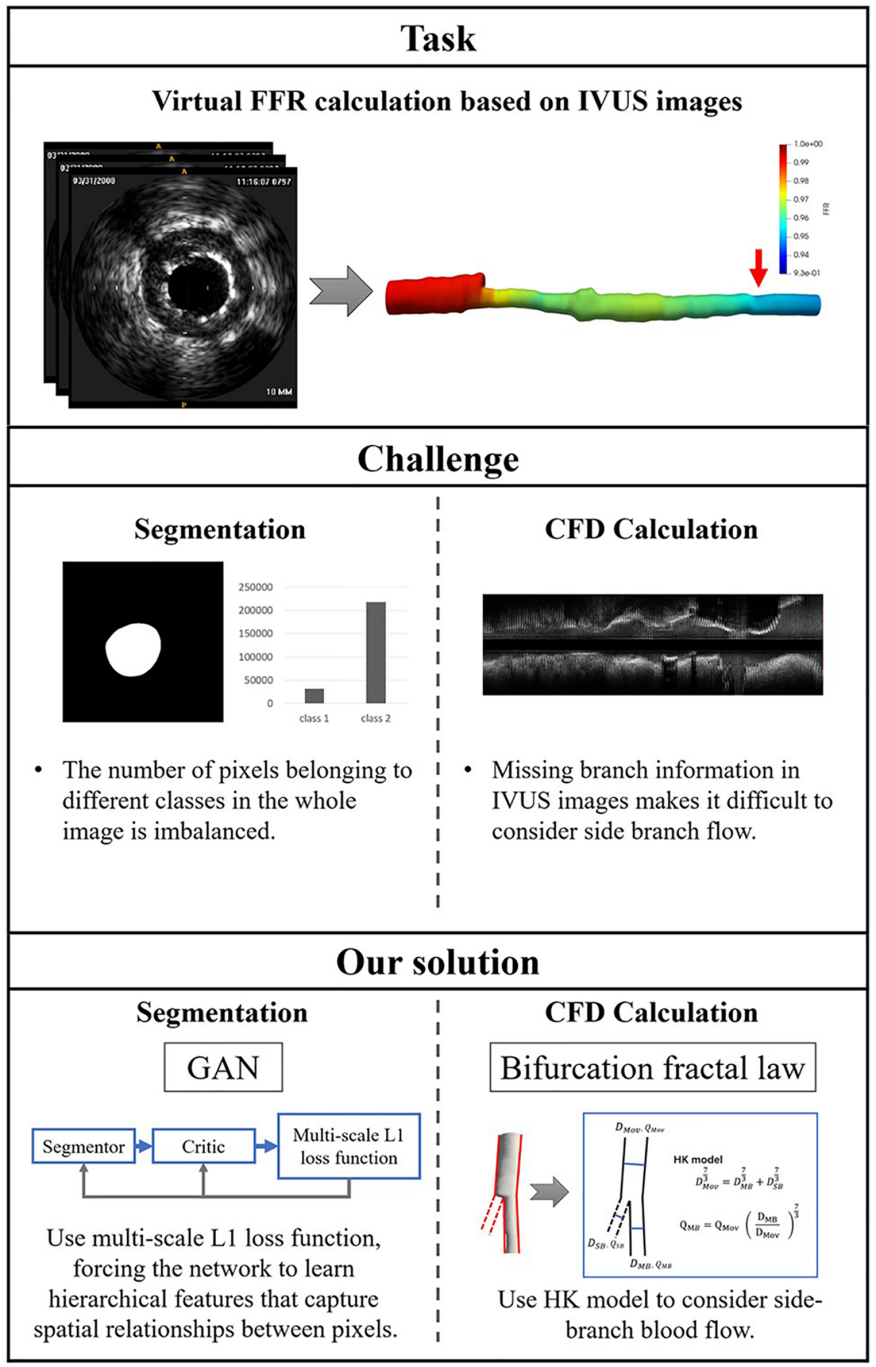
The challenges addressed by this study. The task of this study is to calculate virtual FFR value based on IVUS images. There are a few challenges in this task: First, the challenges of IVUS segmentation are learning contextual relationships between pixels and solving the problem of class imbalance. Second, missing branch information in IVUS images makes it difficult to consider side branch blood flow. We proposed an IVUS-FFR analysis based on generative adversarial network and bifurcation fractal law to address these challenges.

There are two challenges in IVUS-based virtual FFR. First, segmentation of IVUS images requires learning the contextual relationships between pixels and solving the problem of class imbalance. Previous studies primarily used pixel-wise loss in the last layer of their segmentation networks, which may have ignored the features of contextual relationships between pixels. Some studies (15–17) have improved the ability of networks to learn contextual relationships by training CNNs on image patches and using CNNs with various input resolutions or different CNN architectures. However, it is still constrained by the pixel-wise loss and unable to compel the network to learn multi-scale contextual relationships in an end-to-end process. Some studies (18) used skip connections to enable the network to learn contextual relationships directly from the entire image. These studies also used the weighted cross-entropy loss to address the issue of class imbalance brought on by learning from the entire image. This approach suffers from the problem that hyperparameter selection is task-specific and difficult to optimize. Second, missing branch information in IVUS images makes it challenging to consider side branch blood flow. The reconstructed 3D vessels based on IVUS images are single-tube models. The virtual FFR analysis based on this single-tube model is inaccurate as it ignores the effect of side branch blood flow on the lesion vessel blood flow (19). Some studies have manually added the branching orifice model to the reconstructed single-tube vessel model (10, 11), which is more difficult to operate and time-consuming for virtual FFR analysis. Figure 1 illustrates the challenges addressed by this study.

We proposed an IVUS-FFR analysis base on the generative adversarial network (GAN) and bifurcation fractal law. It uses GAN to segment the vessel lumen contours from IVUS images and considers the effect of side branch blood flow by bifurcation fractal law, and finally solves Navier-Stokes equations to obtain virtual FFR values. This method can reduce the manual analysis operation and improve efficiency and clinical feasibility. This paper aims to evaluate the diagnostic performance of the IVUS-FFR analysis, using the invasive FFR as a reference.

## 2. Methods

### 2.1. Study design and study population

**Figure 2:**
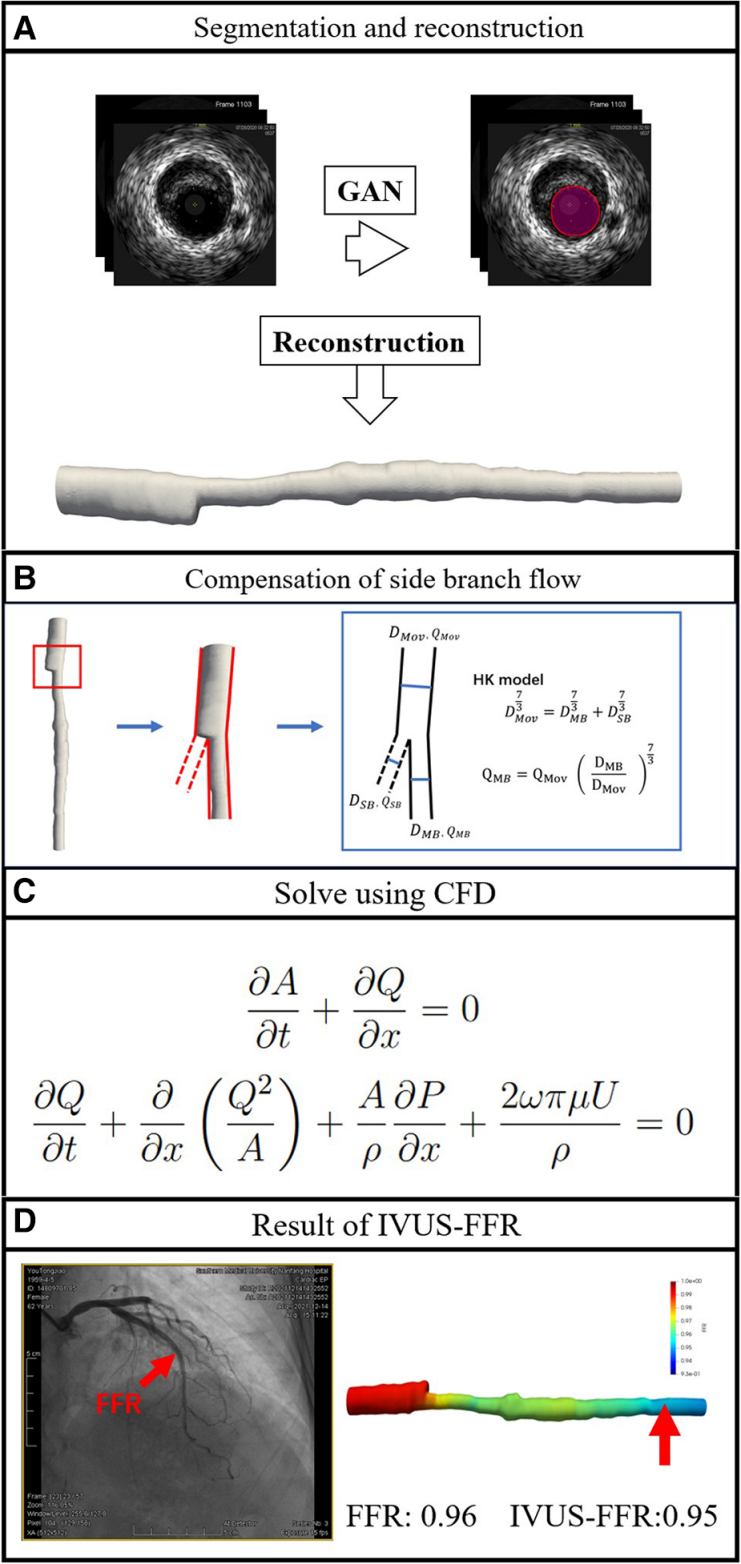
Workflow of IVUS-FFR analysis. The lumen borders in the IVUS images are automatically segmented and reconstructed into a single-tube 3D coronary artery model (**A**). Compensation of branch vessel flow was calculated by the HK model (**B**). Using computational fluid dynamics to solve the Navier-Stokes equations that govern blood flow (**C**). IVUS-FFR was calculated and superimposed on the reconstructed 3D model (**D**).

This is a single-center retrospective study designed with the primary objective of evaluating the diagnostic performance of IVUS-FFR analysis. Data for this study were obtained from the 7th People’s Hospital of Zhengzhou, China between 2020 and 2022. Inclusion criteria: both IVUS and invasive FFR were performed; no less than one diseased coronary artery with 30–90% (visual estimation) stenosis from angiography. Exclusion criteria: age ≥80; coronary artery bypass grafting; IVUS pullback not covering the entire lesion; presence of vasospasm or injury. The study protocol was approved by the Institutional Review Board. Study data were desensitized and personal information was anonymized. Therefore the study does not exceed the minimum risk and the informed consent requirement was waived. Figure 2 shows the workflow of IVUS-FFR analysis.

### 2.2. Coronary angiography and invasive FFR measurement

Coronary angiography was performed via femoral or radial artery access. According to Judkins’ method (20), two different angiographic view angles of each major coronary artery were examined and analyzed. FFR measurements were performed using guide catheters and pressure wires. The aortic pressure at the coronary ostium and the intra-coronary pressure at the distal end of the target lesion were measured under adenosine. invasive FFR value is defined as the ratio of distal lesion pressure to aortic pressure.

### 2.3. IVUS imaging

IVUS images were obtained using the VOLCANO ultrasound detection system or the Boston Scientific ultrasound detection system. The ultrasonic detection probe was placed at the distal end of the diseased blood vessel (along the direction of the guiding wire), and the ultrasonic probe was withdrawn from the distal to the proximal at the speed of 1 mm/s. After continuous image collection and storage, it was to be analyzed offline.

### 2.4. IVUS-FFR analysis

IVUS-FFR analysis was performed by an analyst who was blinded to the invasive FFR values. The entire IVUS-FFR analysis process is divided into two parts: Segmentation of IVUS images and IVUS-FFR Calculation.

#### 2.4.1. Segmentation of IVUS images

**Figure 3:**
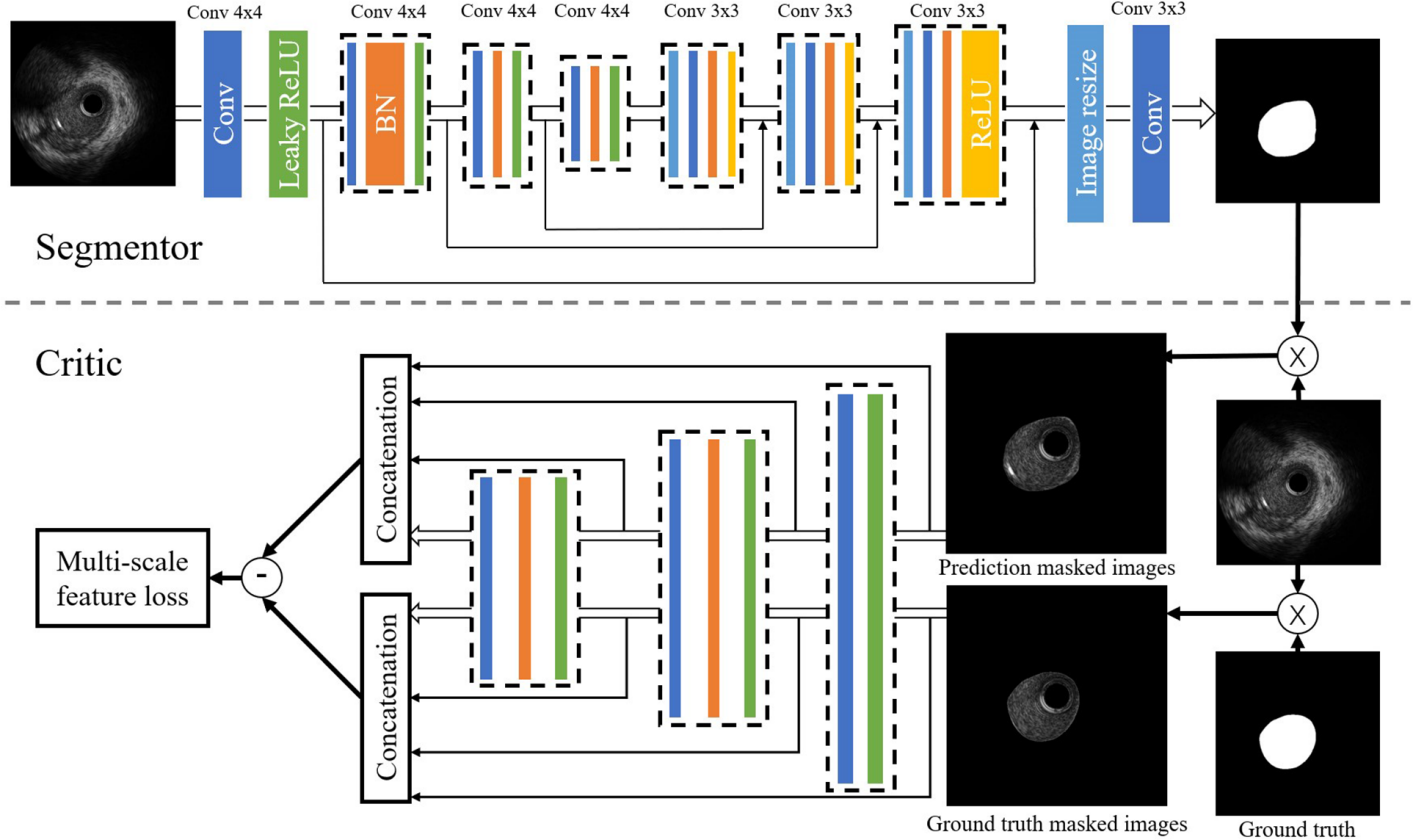
Structure of the SegAN. Segmentor uses the convolutional layer with kernel size 4×4 and stride 2 for downsampling and performs upsampling by convolutional layer with kernel size 3×3 stride 1. Skip connections are added between corresponding layers in the encoder and the decoder. The structure of the critic is similar to the encoder in the segmentor. Hierarchical features are extracted from multiple layers of C and used to compute the multi-scale L1 loss.

We performed the arterial lumen contours segmentation from IVUS images using a generative adversarial network called SegAN (21). SegAN is a high-performance semantic segmentation network. The structure of the network is shown in Figure 3. The network is divided into two parts: Segmentor network (S) and Critic network (C). The segmentor network S is a fully convolutional encoder-decoder structure. Skip connections are also added between corresponding layers in the encoder and the decoder according to the U-net structure. The structure of critic network C is similar to the encoder of S. Hierarchical features are extracted from the multilayers of C and used to compute the multiscale L1 loss. After providing the network with an IVUS image that has been delineated by an experienced IVUS analyst (Ground truth), the network trains both the critic network and the segmentor network using a multiscale L1 loss function:

(1) $$L(\theta_{S},\theta_{C})=\frac{1}{N}\sum_{n=1}^{N}l_{mae}\left\{f_{C}\left[x_{n}\times S\left(x_{n}\right)\right],f_{C}\left(x_{n}\times y_{n}\right)\right\}$$

where N is the number of training images, $x_{n}\times S(x_{n})$ and $x_{n}\times y_{n}$ are prediction masked images and ground truth masked images, respectively. $f_{c}(x)$ indicates Critic’s hierarchical feature extraction of the input image x. $l_{mae}$ is the Mean Absolute Error (MAE). More specifically, the $l_{mae}$ function is defined as:

(2) $$l_{mae}[f_{C}(x),f_{C}(x^{\prime })]=\frac{1}{L}\sum_{i=1}^{L}\|f_{C}^{i}(x)-f_{C}^{i}(x^{\prime }){\|}_{1}$$

where L is the total number of layers in Critic. $f_{C}^{i}(x)$ is the feature extracted for input x at layer i.

During the end-to-end training process, the segmentor is trained to minimize the loss function with the goal of reducing the difference between the output image and the ground truth, while the critic aims to maximize this loss function as a way to distinguish the segmentor’s output image from the ground truth. After training with 270,362 IVUS images, the segmentor can generate images that are very close to the ground truth. After the segmentation of IVUS images, a single-tube 3D coronary artery model is reconstructed based on the results of segmentation.

#### 2.4.2. IVUS-FFR calculation

The four steps for calculating IVUS-FFR are as follows: Extraction of centerline and cross-sectional area; Centerline model splitting; Stenosis detection; and IVUS-FFR calculation of each segment.

Firstly, the centerline and cross-sectional area of the vessel are extracted based on the reconstructed 3D coronary model simultaneously, and the branch nodes connecting the side branches of the vessels are labeled according to the IVUS images. Secondly, the centerline is split into normal segments and junctions at the labeled branch nodes. Thirdly, a stenosis detection algorithm that compares the difference between the radius of the target vessel and the healthy vessel at the same location in a normal person, is applied to automatically detect the location of the stenotic portion in the centerline model and define the stenosis segment (22). Fourthly, to determine the pressure distribution throughout the vessel, the Navier-Stokes equations for each segment are sequentially solved using the centerline model and the cross-sectional area (23–25). In particular, calculations at the junctions are done according to the HK model (26): 1, measure the diameters at both ends of the junction (i.e., the diameter of the mother vessel $D_{Mov}$ and the diameter of the main branch $D_{MB}$), and 2, substitute into HK model to calculate the flow variation ($Q_{Mov}$ is calculated from the previous segment). Thus achieving the purpose of accurate IVUS-FFR calculations in a single-tube coronary artery model.

Accurate boundary conditions are one of the central parameters to obtain accurate simulations (27–29). During the IVUS-FFR calculation, we set pressure and blood velocity at the vessel inlet. Where the inlet pressure Pa is set as the mean aortic pressure. The inlet blood velocity V is set as the hyperemic flow rate, which is determined by first using the TIMI framing method to measure the resting flow rate (30) and then converting it to the hyperemic flow rate according to a specific empirical formula (31). The outlet boundary condition is assumed to be a fully developed flow. The calculation process is carried out automatically and the virtual FFR value is calculated for each point in the centerline based on the final pressure distribution field obtained. To investigate the diagnostic performance and computation time of IVUS-FFR, we performed a comparison experiment using FFR-branch. In earlier studies (10, 11), FFR-branch was commonly used for virtual FFR analysis. In contrast to considering branch flow using the bifurcation fractal law, this method considers branch flow by manually extending the side branch from the bifurcation ostia and subsequently performing CFD calculations using the modified model as the reference lumen.

More details of the IVUS-FFR analysis can be found in “DETAILS OF IVUS-FFR ANALYSIS” section in the *Supplementary Material*.

### 2.5. Statistical analysis

The quantitative variables were the mean ± SD of normally distributed variables and the median of Variables with non-normal distribution (interquartile variance [IQR]). Categorical variables are expressed as quantities (percentages). IVUS-FFR and invasive FFR were considered as continuous variables and were classified as dichotomous variables with a threshold of 0.80 (FFR≤0.80 was considered as myocardial ischemia). The analysis is done on a per-vessel basis. The agreement of IVUS-FFR with invasive FFR was assessed using the Bland-Altman method. The correlation between IVUS-FFR and invasive FFR was determined by the Pearson correlation coefficient (r). The diagnostic performance of IVUS-FFR was evaluated using invasive FFR as a reference. The area under the ROC (AUC) was used to estimate the diagnostic performance of the method. All statistical analyses were performed using MedCalc v19.7 (MedCalc, Belgium) and SPSS software v26.0 (IBM, US).

## 3. Result

### 3.1. Baseline clinical characteristics

In all, 92 patients with 113 vessels were included in this study, among whom 5 patients with 5 vessels were excluded according to the exclusion criteria mentioned in Method. The final study population comprised 87 patients with 108 vessels. The baseline clinical characteristics and vessel characteristics are listed in Table 1. The average times for IVUS-FFR and FFR-branch were 3 and 20 min, respectively.

**Table 1:** Baseline clinical characteristics.

Patient characteristics	n=87
Age (years)	60.6±9.7
Male	66 (76)
Body surface area	1.71±0.11
Heart rate (bpm)	68.4±4.8
SBP (mmHg)	129.5±15.1
DBP (mmHg)	77.9±8.9
Hypertension	55 (63)
Hyperlipidemia	61 (70)
Prior MI	3 (3)
Vessel characteristics	n=108
LAD	67 (62)
LCX	15 (14)
RCA	26 (24)

Data are presented as mean ± SD or number (%), as appropriate. DBP Diastolic blood pressure, SBP systolic blood pressure, MI myocardial infarction, LAD left anterior descending artery, LCX left circumflex artery, RCA right coronary artery.

### 3.2. Effectiveness of SegAN

**Figure 4:**
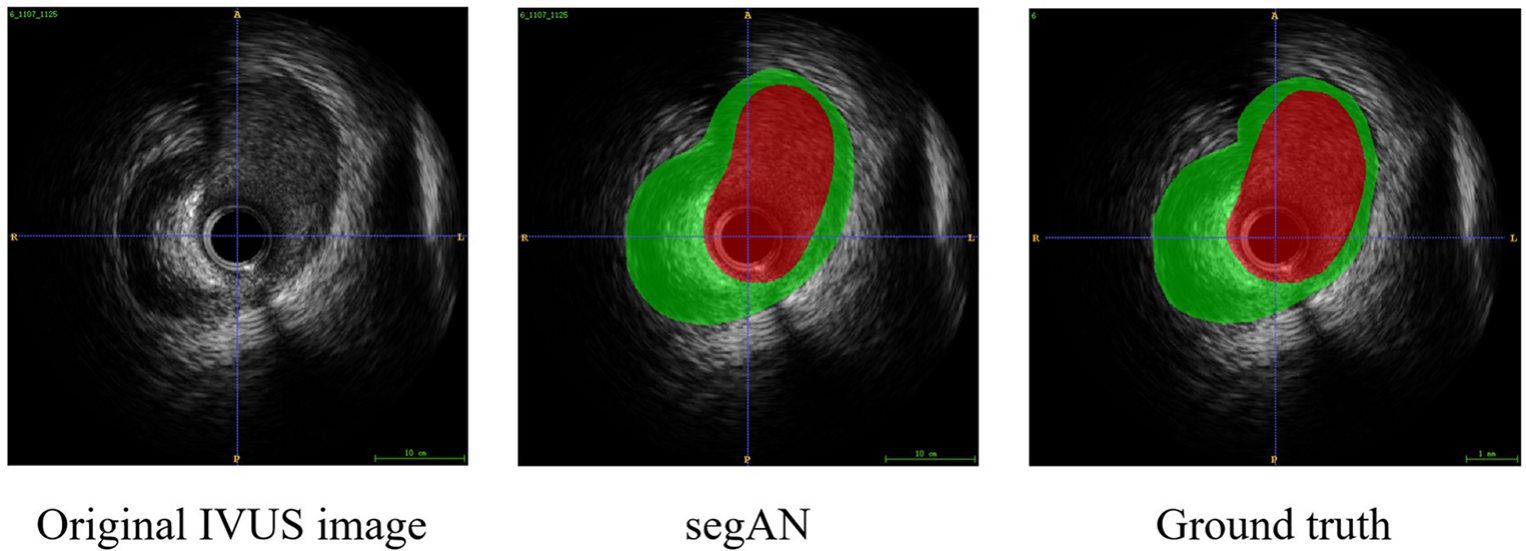
Segmentation result of SegAN compared with the Ground truth. From left to right are the original IVUS image, the segmentation result of SegAN, and the Ground truth, respectively. In the figure, the red region is the lumen contour and the green region is the MA contour. It can be seen that the segmentation result of SegAN is very similar to the ground truth, and the edges are smooth.

To validate the effectiveness of SegAN, we used three indexes to evaluate the similarity of SegAN segmentation results and ground truth. The three indexes include Dice index, Hausdorff index, and Jaccard index. We compared the performance of SegAN and other state-of-the-art methods for segmenting MA contours and lumen contours from IVUS images. As shown in Table 2, the SegAN approach has better segmentation performance than the other state-of-the-art methods. Figure 4 shows the segmentation results of SegAN compared with the ground truth, where the red region is the MA contour and the green region is the lumen contour. The results indicated that the segmentation result of SegAN is very close to the ground truth and has smooth edges. In summary, the SegAN segmentation network used in this study is effective. For more detailed validation, please see the “GENERALIZATION PERFORMANCE OF SEGAN” and “IMPACT OF ACCURATE SEGMENTATION FOR IVUS-FFR” sections in the *Supplementary Material*.

**Table 2:** Comparison of SegAN and state-of-the-art methods.

	Dice		Hausdorff		Jaccard	
	Lumen	MA	Lumen	MA	Lumen	MA
DPUNet (32)	0.902	0.880	5.02	9.38	0.837	0.800
mfaUNet (33)	0.912	0.923	4.18	4.49	0.851	0.864
MPUNet (34)	0.907	0.896	4.61	7.77	0.844	0.823
FRRNet (35)	0.858	0.839	6.69	11.8	0.767	0.737
ivusNet (18)	0.903	0.893	5.12	8.53	0.838	0.818
HRNet (36)	0.901	0.914	4.90	6.04	0.835	0.851
CapsNet (37)	0.877	0.849	7.27	11.8	0.799	0.753
HNF3-Net (38)	0.917	0.919	3.61	5.15	0.861	0.859
Unet (39)	0.892	0.893	5.32	7.56	0.822	0.819
SMP (28)	0.925	0.931	3.47	3.93	0.873	0.879
SegAN	0.953	0.965	2.27	2.41	0.913	0.935

### 3.3. Comparison of IVUS-FFR and FFR-branch

To demonstrate that IVUS-FFR with bifurcation fractal law can be an alternative to FFR-branch, we performed virtual FFR analysis based on these two different methods (based on the same patient data and the same boundary conditions) and compared the results.

**Figure 5:**
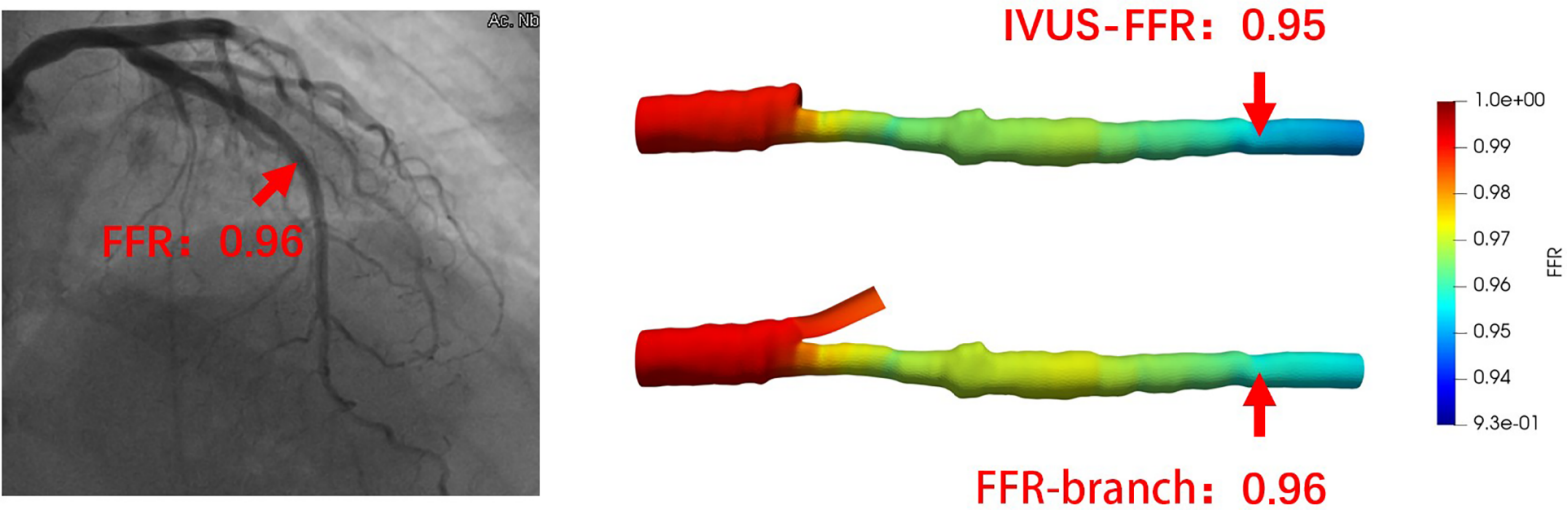
Examples of the analysis results for IVUS-FFR and FFR-branch. The image on the left shows the angiography image of the patient and the invasive FFR markers at the location of the lesion. The right panel shows the results of the IVUS-FFR and FFR-branch, respectively. It can be seen that IVUS-FFR is in agreement with the invasive FFR and FFR-branch.

**Figure 6:**
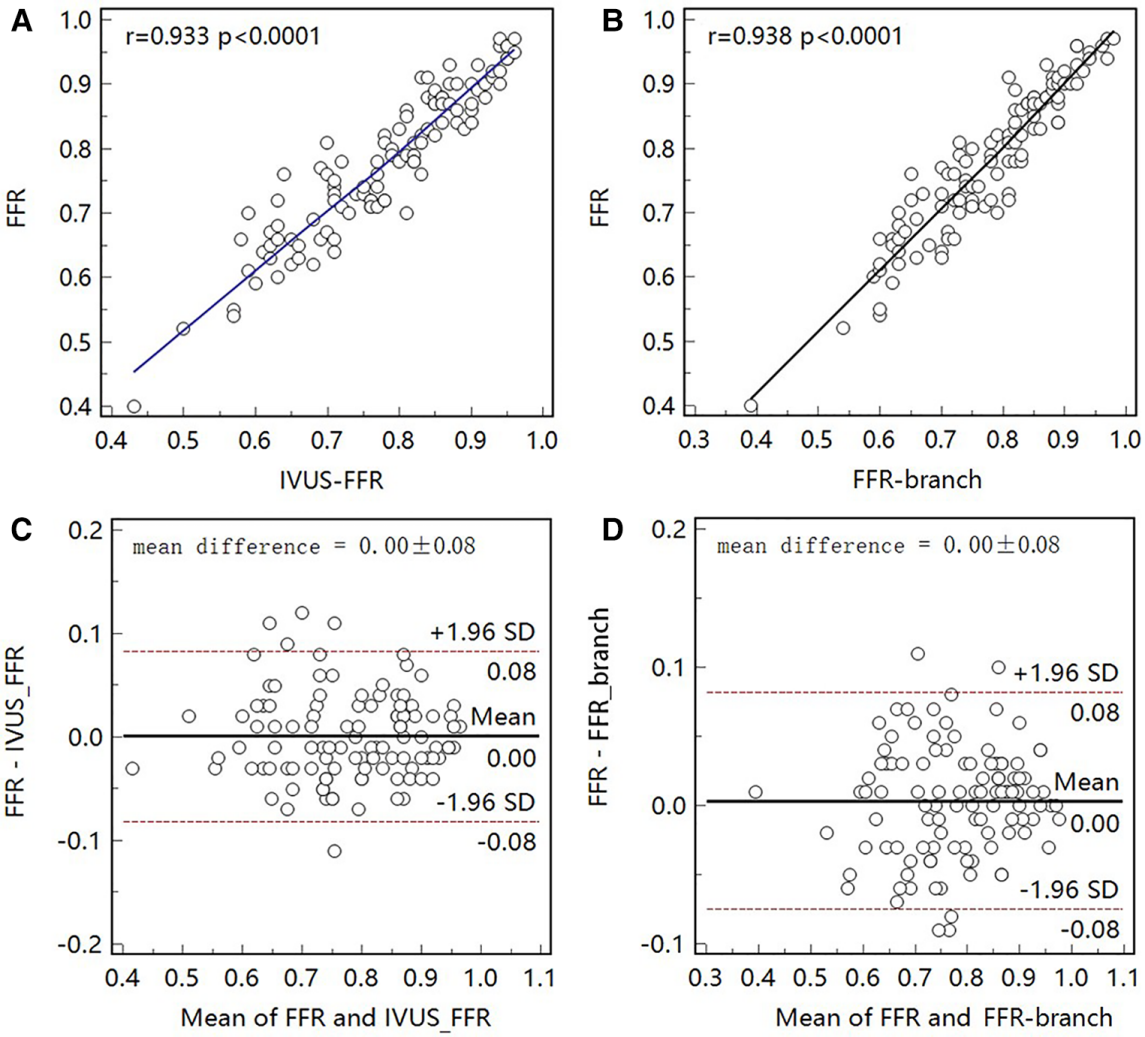
Correlation and agreement between invasive FFR and IVUS-FFR/FFR-branch. (**A**, **B**) Strong correlation (r=0.933, 0.938) between invasive FFR and IVUS-FFR/FFR-branch was observed. (**C**, **D**) Bland-Altman plots show good agreement between invasive FFR and IVUS-FFR/FFR-branch.

**Figure 7:**
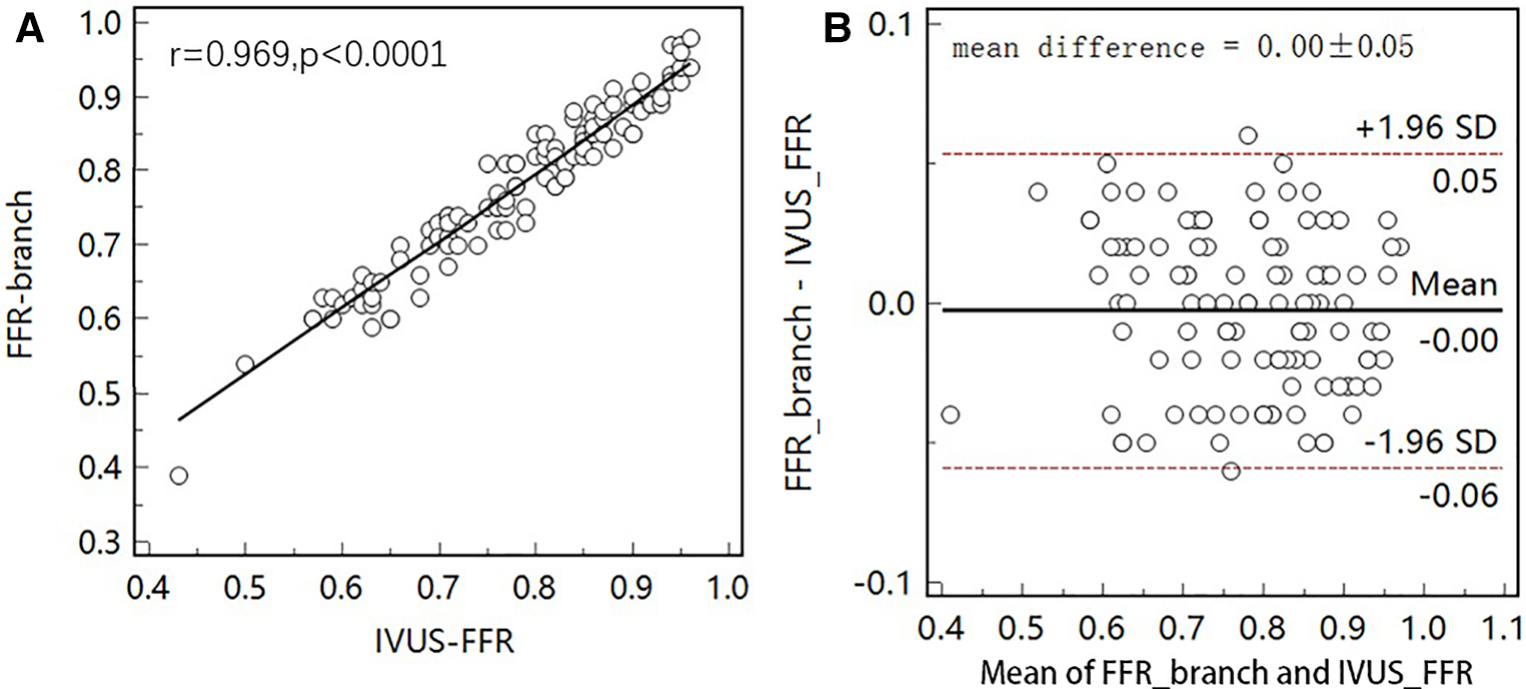
Correlation and agreement between IVUS-FFR and FFR-branch. (**A**) Strong correlation (r=0.969) between IVUS-FFR and FFR-branch was observed. (**B**) Bland-Altman plot shows good agreement between IVUS-FFR and FFR-branch.

**Figure 8:**
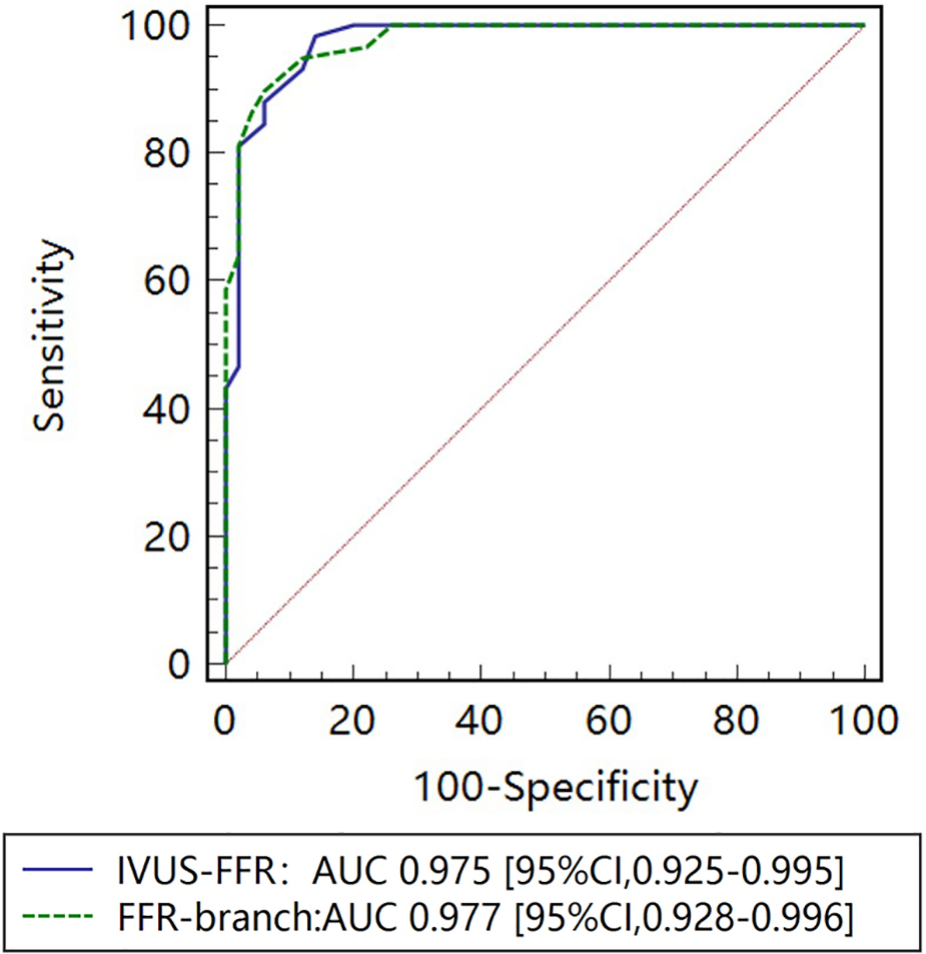
Receiver operating characteristic (ROC) curves for diagnosis of physiologically significant stenosis. The diagnostic performance of IVUS-FFR is very close to that of the FFR-branch. AUC indicates the area under the curves.

Figure 5 shows examples of the analysis results for IVUS-FFR and FFR-branch, where IVUS-FFR is 0.95, and FFR-branch is 0.96. Figure 6 shows the scatter plots and Bland-Altman plots for invasive FFR and IVUS-FFR/FFR-branch. The median values of invasive FFR, IVUS-FFR, and FFR-branch were 0.785 (0.753–0.820), 0.795 (0.763–0.827), and 0.790 (0.750–0.820), respectively. A strong correlation with invasive FFR can be observed for both methods (IVUS-FFR: r=0.933, p<0.0001; FFR-branch: r=0.9377, p<0.0001). It can be seen from the Bland-Altman plots that both IVUS-FFR and FFR-branch are in good agreement with invasive FFR. And, there is also a good correlation and agreement between the two methods (r=0.969, p<0.0001, Figure 7). Table 3 demonstrates the diagnostic performance of both methods (with invasive FFR≤0.80 as the disease benchmark). The ROC curve (Figure 8) was plotted and AUC was 0.975 (95% IC: 0.925–0.995) for IVUS-FFR and 0.977 (95% IC: 0.928–0.996) for FFR-branch.

**Table 3:** Diagnostic Performance of IVUS-FFR and FFR-branch to identify invasive FFR ≤ 0.80.

	IVUS-FFR ≤ 0.80	FFR-branch ≤ 0.80
Accuracy, % (95% CI)	90.7 (83.6–95.5)	91.7 (84.8–96.1)
Sensitivity, % (95% CI)	89.7 (78.8–96.1)	89.7 (78.8–96.1)
Specificity, % (95% CI)	92.0 (80.8–97.8)	94.0 (83.5–98.7)
Positive predictive value, % (95% CI)	92.9 (83.4–97.1)	94.5 (85.2–98.1)
Negative predictive value, % (95% CI)	88.5 (78.2–94.3)	88.7 (78.5–94.4)
Positive likelihood ratio (95% CI)	11.2 (4.36–28.8)	14.9 (4.97–44.9)
Negative likelihood ratio (95% CI)	0.112 (0.052–0.241)	0.110 (0.051–0.236)

The calculation of IVUS-FFR utilizes the HK model to account for the effect of side branch blood flow.

### 3.4. Effect of bifurcation fractal law on IVUS-FFR

**Figure 9:**
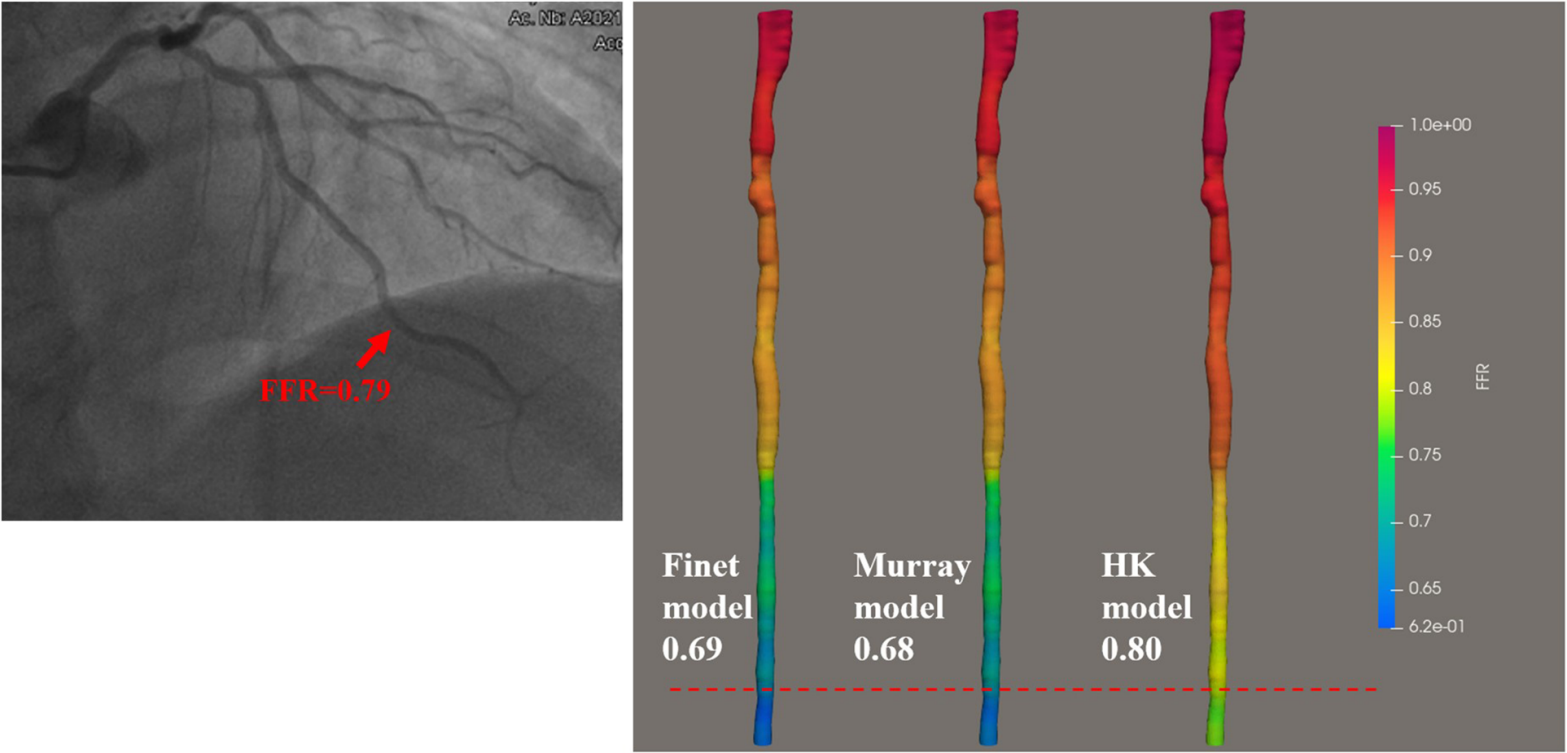
Examples of the calculated results for the Finet model, Murray model, and HK model. The image on the left shows the angiography image of the patient and the invasive FFR markers at the location of the lesion. The right panel shows the calculated results of the three models, respectively. It can be seen that the HK model has the highest accuracy.

There are three bifurcation fractal laws that are currently used to consider the effects of side branch blood flow, namely the HK model (26), the Finet model (40), and the Murray model (41). In this study, IVUS-FFR analysis was performed according to three models (based on the same patient data and the same boundary conditions), and finally, the effects of the three models on the accuracy of IVUS-FFR analysis were compared. Figure 9 shows examples of the analysis results for the three models.

**Figure 10:**
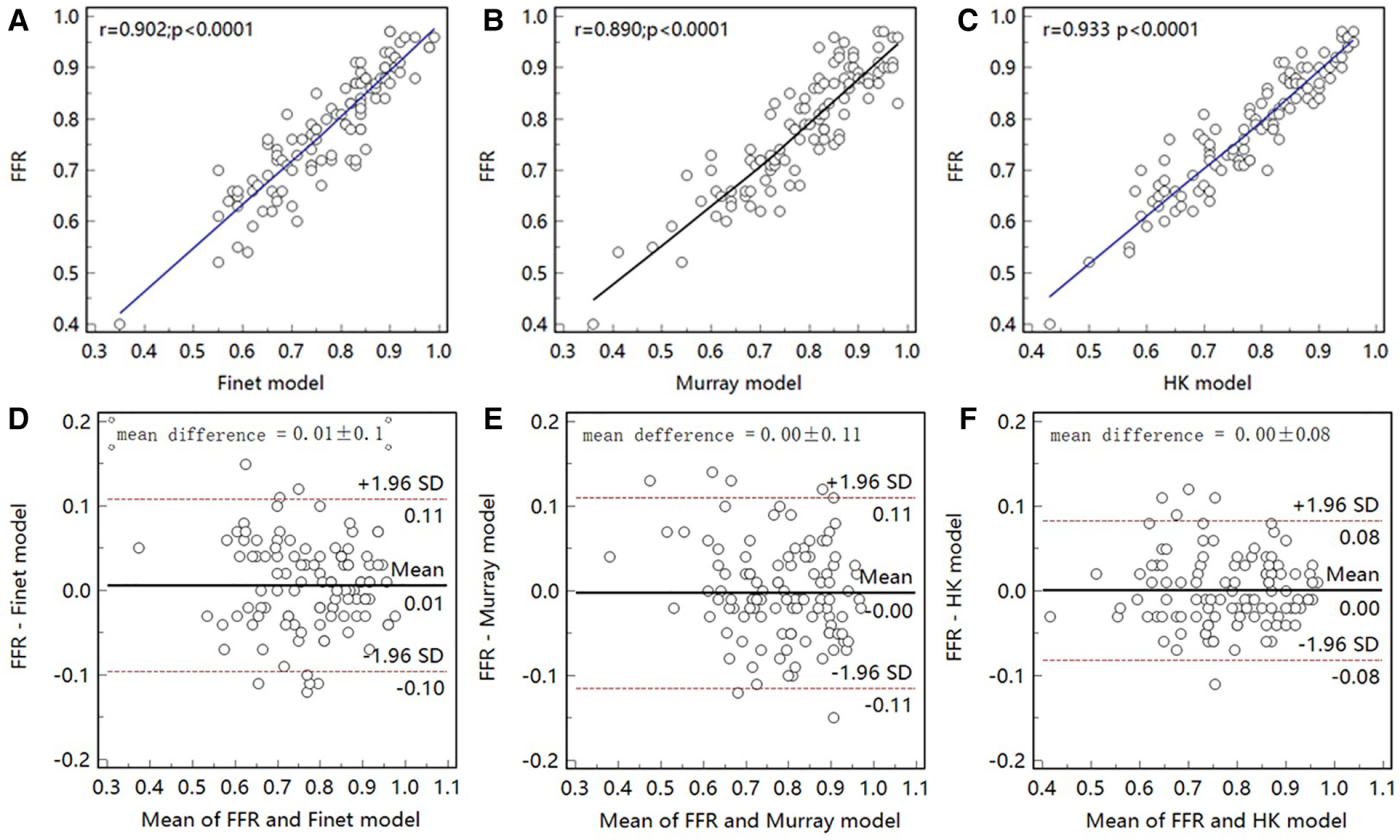
Correlation and agreement between Finet model/Murray model/HK model and invasive FFR. (**A**–**C**), The correlation between HK model and invasive FFR (r=0.933) was higher than that between Finet model (r=0.902) and Murray model (r=0.890). (**D**–**F**) The HK model has the highest agreement with invasive FFR.

**Figure 11:**
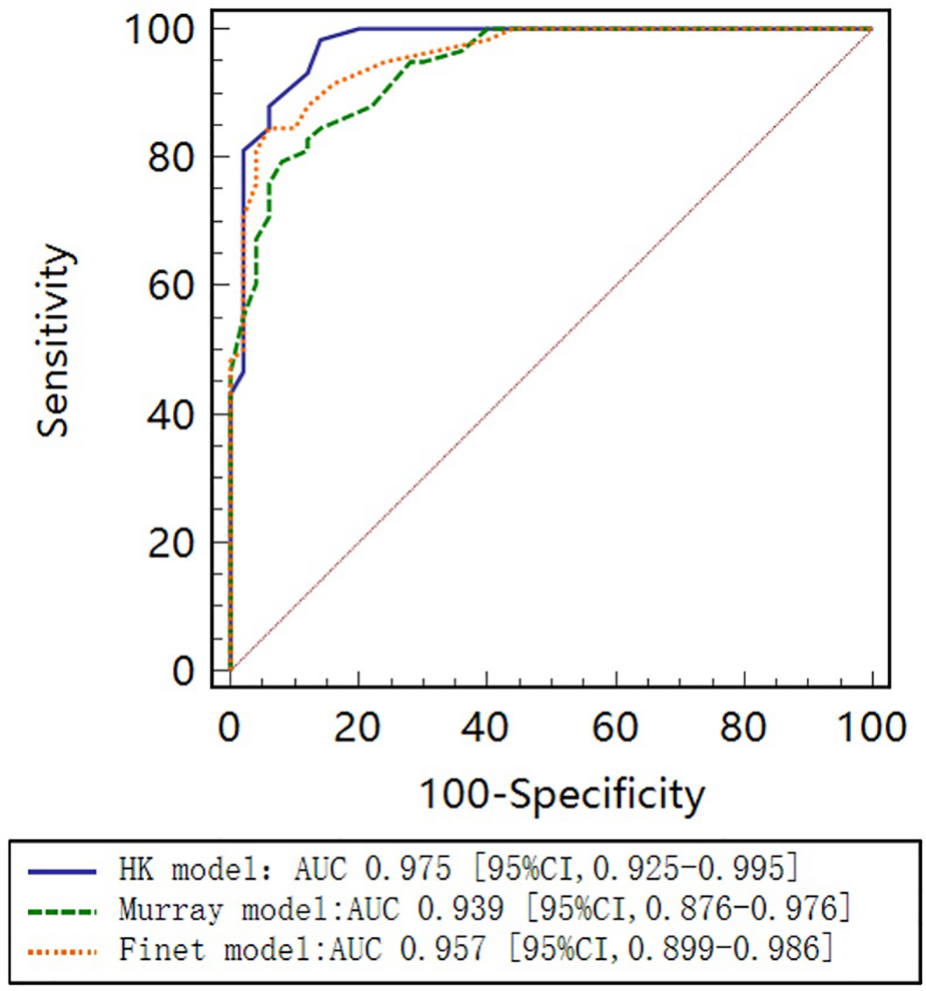
Receiver operating characteristic (ROC) curves for diagnosis of physiologically significant stenosis. HK model shows higher diagnostic accuracy than Finet model and Murray model.

As seen in the correlation and agreement analysis (Figure 10), the HK model had the highest correlation (r=0.9328; p<0.0001) and agreement with invasive FFR among the three models, and the Murray and Finet models had poorer correlation and agreement with invasive FFR. Table 4 shows the diagnostic performance of the three models (with invasive FFR≤0.80 as the disease benchmark). From the ROC curve (Figure 11), it can be seen that the HK model has the best performance with an area under the curve (AUC) of 0.975 (95% IC: 0.925–0.995), and the Murray and Finet models have poor performance with an area under the curve of 0.939 (95% IC: 0.876–0.976) and 0.957 (95% IC: 0.899–0.986), respectively.

**Table 4:** Diagnostic performance of HK model, Finet model, and Murray model to identify invasive FFR ≤ 0.80.

	HK model	Finet model	Murray model
Accuracy, % (95% CI)	90.7 (83.6–95.5)	87.0 (79.2–92.7)	85.2 (77.1–91.3)
Sensitivity, % (95% CI)	89.7 (78.8–96.1)	84.5 (72.6–92.7)	82.8 (70.6–91.4)
Specificity, % (95% CI)	92.0 (80.8–97.8)	90.0 (78.2–96.7)	88.0 (75.7–95.5)
Positive predictive value, % (95% CI)	92.9 (83.5–97.1)	90.7 (80.9–95.8)	88.9 (78.9–94.5)
Negative predictive value, % (95% CI)	88.5 (78.2–94.3)	83.3 (73.1–90.2)	81.5 (71.3–88.6)
Positive likelihood ratio (95% CI)	11.2 (4.36–28.8)	8.4 (3.65–19.5)	6.90 (3.23–14.7)
Negative likelihood ratio (95% CI)	0.112 (0.052–0.241)	0.172 (0.094–0.317)	0.196 (0.110–0.348)

In order to compare the effects of different bifurcation fractal laws, the HK model, Finet model, and Murray model were used to calculate IVUS-FFR, respectively.

## 4. Discussion

In this study, we proposed an IVUS-FFR analysis using generative adversarial networks to segment IVUS images and consider side branch blood flow by the bifurcation fractal law. We assessed the effectiveness of applying SegAN to IVUS image segmentation and contrasted the diagnostic performance of IVUS-FFR with that of invasive FFR and FFR-branch. The results showed that IVUS-FFR and invasive FFR had a good correlation and agreement. Additionally, IVUS-FFR requires significantly less computation time while maintaining the same level of diagnostic performance as FFR-branch. We also investigated how three hydrodynamic models affected the IVUS-FFR analysis’ accuracy, and the results revealed that the HK model has the highest accuracy and is most suitable for calculating the IVUS-FFR.

The dimension of the arterial lumen is a major determinant of resistance to flow in coronary vessels (10). Therefore, precise segmentation of IVUS images is a central feature of IVUS-FFR (42, 43). The method proposed in this study uses a modified generative adversarial network, SegAN, to segment IVUS images. As opposed to classical GAN, which trains the generator and discriminator using different loss functions (44), SegAN trains the segmentor and critic networks using a multi-scale L1 loss function. This enables SegAN to be trained end-to-end on the entire image without patches sampling or inputting images in various resolutions. Similar to the idea of sampling multiple times in the same image in patch training (45), the critic network considers the feature differences between predicted segmentation and ground truth at multiple scales (i.e., considering differences in multiple layers) utilizing the multi-scale L1 loss function. Gradients that flow through the critic are then used to train the segmentor. This approach is used to force the network to learn the contextual relationships between pixels in an end-to-end process and to avoid the class imbalance problem. Thus, SegAN is more suitable for segmenting IVUS images than previous methods.

During virtual FFR analysis, the blood flow in the target diseased vessel is affected by the side branch blood flow (14, 19, 46). In our proposed method, the effect of side branch blood flow is considered using the bifurcation fractal law. It is only required to measure the diameters at both ends of the lesioned vessel branches to calculate the flow variation, thus achieving the purpose of accurate IVUS-FFR analysis in a single-tube coronary artery model. Compared with previous studies (10, 11), which were mainly based on the extension of side branches from the bifurcation, the method proposed in this study shortens the analysis time while maintaining accuracy.

Different bifurcation fractal laws yield different estimates of side branch blood flow (47). The accuracy of IVUS-FFR can be increased by locating hydrodynamic models that can precisely assess the flow relationship between the coronary arteries’ main branches and side branches. Currently, three bifurcation fractal laws are applied to calculate the compensation of branched flow: Murray, Finet, and HK. In this study, IVUS-FFR was calculated on the same data according to three models separately and their diagnostic performance was evaluated. The final experimental results show that the HK model has the best diagnostic performance. This is likely because the steady-state assumptions of the HK model are more applicable to obtaining blood flow relationships between microvessels (48).

The current study has some inherent limitations. First, coronary circulation can be impacted by conditions like severe LV hypertrophy, hypertrophic cardiomyopathy, severe systolic dysfunction, infarct-related arteries, or significant valvular disease. The diagnostic performance of the IVUS-FFR was not validated in cases with these diseases, which may affect the accuracy of the results of this study. Second, the method used in this study is limited by the quality of the IVUS images, which can affect the accuracy of the results if the IVUS images are of poor quality. Methods to reduce the impact of IVUS image quality on IVUS-FFR accuracy are to be investigated in future studies.

## 5. Conclusion

This study demonstrated that our IVUS-FFR analysis correlates and agrees well with invasive FFR, showing good diagnostic performance. Compared to FFR-branch, IVUS-FFR has the same level of diagnostic performance and significantly lower computation time. The time-saving and high accuracy characteristics show the potential of applying IVUS-FFR to a wide range of applications in catheterization laboratories.

## Data Availability

The raw data supporting the conclusions of this article will be made available by the authors, without undue reservation.
