# Supplementary material for: Diagnostic performance of IVUS-FFR analysis based on generative adversarial network and bifurcation fractal law for assessing myocardial ischemia
Source: Front Cardiovasc Med. 2023 Mar 20;10:1155969. doi: 10.3389/fcvm.2023.1155969 (PMC10067879; doi:10.3389/fcvm.2023.1155969)
Supplement: Supplementary file 1 [file Datasheet1.pdf]

Supplementary Material

DETAILS OF IVUS-FFR ANALYSIS

For the extracted centerline model, which has been divided into several segments, Navier-Stokes equations that govern the blood flow are applied to calculate the pressure distribution in each of them(1, 2, 3). For all segments, the governing equations are as follows:

$$\frac{\partial Q}{\partial x} = 0 \tag{S1}$$

$$\frac{\partial}{\partial x}\left(\frac{Q^2}{A}\right) + \frac{A}{\rho}\frac{\partial P}{\partial x} = 0 \tag{S2}$$

where Q is the flow rate, A is the lumen cross-sectional area, P is the average pressure in the lumen cross-section, and U = $\frac{Q}{A}$ is the cross-sectional average velocity.

At junctions, we consider the effect of side branch blood flow according to HK model(4):

$$D_{Mov}^{\frac{7}{3}} = D_{MB}^{\frac{7}{3}} + D_{SB}^{\frac{7}{3}} \tag{S3}$$

$$\frac{Q_{MB}}{Q_{Mov}} = \left(\frac{D_{MB}}{D_{Mov}}\right)^{\frac{7}{3}} \tag{S4}$$

where D_{Mov} is the mother vessel, D_{MB} is the main branch, D_{SB} is the side branch, and Q_{MB} and Q_{Mov} are the blood flow of the mother vessel and the main branch, respectively.

For comparative tests, we applied the Murray model(5) and the Finet model(6) on the same data to consider side branch blood flow:

Murray model:

$$D_{Mov}^3 = D_{MB}^3 + D_{SB}^3 \tag{S5}$$

Finet model:

$$D_{Mov} = 0.678(D_{MB} + D_{SB}) \tag{S6}$$

In addition, it is necessary to apply the mass conservation equation and the junction model to calculate the pressure distribution.

$$\sum_{i=1}^{N} Q_i = 0 \tag{S7}$$

$$P_1 + \frac{\rho}{2}U_1^2 = P_i + \frac{\rho}{2}U_i^2 \quad i = 2, ..., N \tag{S8}$$

where N is the number of converging segments. Note that i = 1 is taken as the supplier branch.

In the stenosis segment, we apply the lumped parameter model proposed in (7) to calculate the pressure distribution. The governing equation for the pressure drop at both ends of the stenosis segment is:

$$\Delta P = K_v \frac{\mu}{D} U + K_t \frac{\rho}{2} \left[\frac{A}{A_S} - 1 \right]^2 U^2 + K_u \rho L_S \frac{\mathrm{d}U}{\mathrm{d}t} \tag{S9}$$

where U and A (D the diameter) are the velocity and lumen area in the unobstructed part of the vessel, L_S is the stenosis length, A_S is the minimum stenosis area, and K_v, K_t and K_u are model parameters characterizing viscous, turbulent and inertial effects, respectively. Specifically, L_S and A_S are characterized by the stenosis detection algorithm.

GENERALIZATION PERFORMANCE OF SEGAN

To investigate the generalization performance of segAN, we inputted IVUS images with different frequency modes (20Hz, 40Hz, 60Hz) to segAN and compared the Dice index, Jaccard index, and Hausdorff index of the three outputs. Table S1 showed the comparison of the three outputs, and Figure S1 showed the segmentation results of IVUS images with three different frequency modes. The comparison results demonstrated that segAN has good segmentation performance for all three different inputs, which indicates that segAN has good generalization performance.

Table S1. Comparison of the segmentation results with different frequency modes of IVUS images.

	Dice		Hausdorff		Jaccard	
	lumen	ma	lumen	ma	lumen	ma
20Hz	0.943	0.960	2.63	3.09	0.895	0.927
40Hz	0.956	0.967	2.25	2.39	0.917	0.937
60Hz	0.962	0.967	2.00	2.41	0.928	0.937

IMPACT OF ACCURATE SEGMENTATION FOR IVUS-FFR

We reconstructed three 3D vascular models using IVUS segmentation results with different degrees of accuracy (based on the same patient). And performed IVUS-FFR calculations on these three models separately. The comparison results (Figure S2) show that the IVUS-FFR calculated using the accurate segmentation is closest to the invasive FFR compared to the inaccurate segmentation, indicating that the accurate segmentation of IVUS images is very important for IVUS-FFR.

REFERENCES

1 .Behr M, Hormes M, Steinseifer U, Arora D, Coronado O, Pasquali M. A review of computational fluid dynamics analysis of blood pumps. *European Journal of Applied Mathematics* **20** (2009) 363–397.

2 .Müller LO, Blanco PJ, Watanabe SM, Feijóo RA. A high-order local time stepping finite volume solver for one-dimensional blood flow simulations: application to the adan model. *International Journal for Numerical Methods in Biomedical Engineering* **32** (2016) e02761.

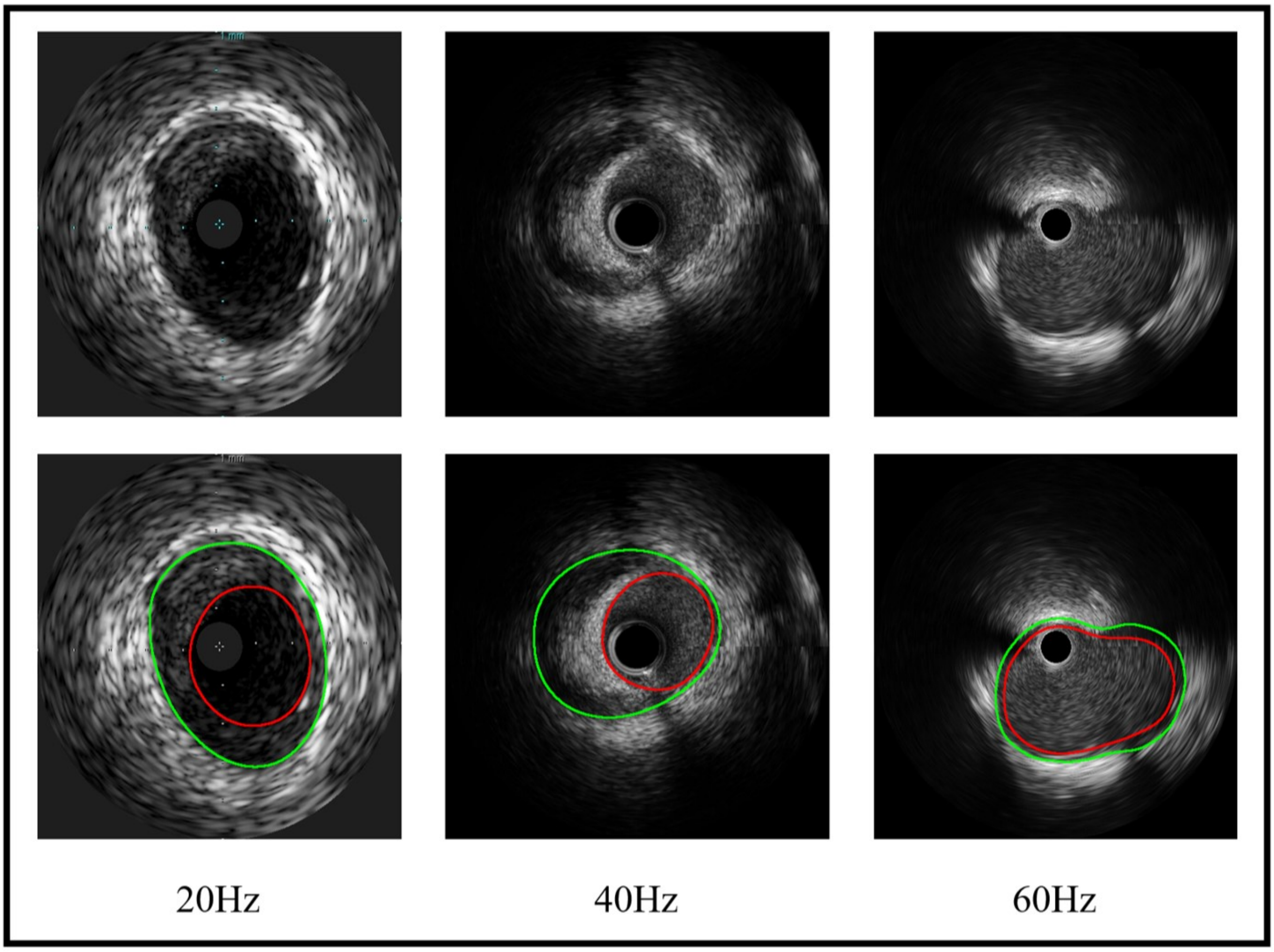

Figure S1. Segmentation results of IVUS images with three frequency modes. From left to right, the IVUS images of three different frequency modes and their segmentation results are shown respectively. It can be seen that segAN has good segmentation performance for all three IVUS images.

3 .Huo Y, Kassab GS. A hybrid one-dimensional/womersley model of pulsatile blood flow in the entire coronary arterial tree. *American Journal of Physiology-Heart and Circulatory Physiology* **292** (2007) H2623–H2633.

4 .Huo Y, Kassab GS. A scaling law of vascular volume. *Biophysical journal* **96** (2009) 347–353.

5 .Murray CD. The physiological principle of minimum work: a reply. *The Journal of general physiology* **14** (1931) 445.

6 .Finet G, Gilard M, Perrenot B, Rioufol G, Motreff P, Gavit L, et al. Fractal geometry of arterial coronary bifurcations: a quantitative coronary angiography and intravascular ultrasound analysis. *EuroIntervention: journal of EuroPCR in collaboration with the Working Group on Interventional Cardiology of the European Society of Cardiology* **3** (2008) 490–498.

7 .Young DF, Tsai FY. Flow characteristics in models of arterial stenoses—ii. unsteady flow. *Journal of biomechanics* **6** (1973) 547–559.

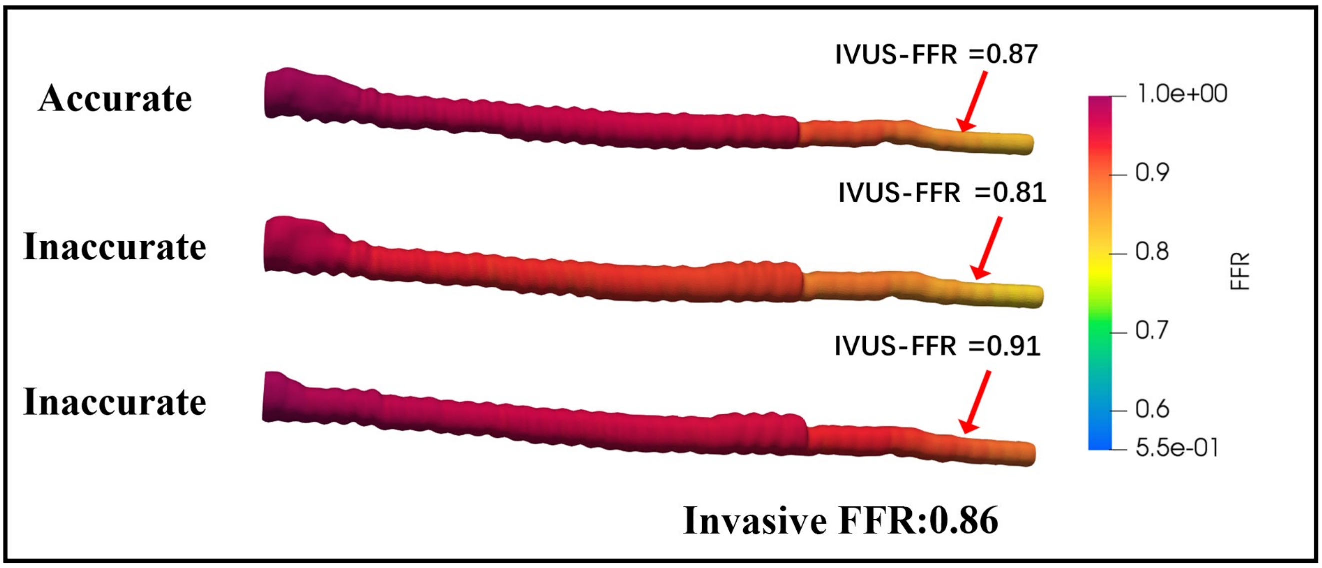

Figure S2. Impact of accurate segmentation for IVUS-FFR. Three IVUS-FFR calculation results of the same patient are shown from top to bottom, each of them was calculated using segmentations of different accuracy degrees. It can be seen that the IVUS-FFR calculated using the accurate model is closest to the invasive FFR compared to the inaccurate model, indicating that the accurate segmentation of IVUS images is very important for IVUS-FFR.
